# Forest Aboveground Biomass Estimation Based on Unmanned Aerial Vehicle–Light Detection and Ranging and Machine Learning

**DOI:** 10.3390/s24217071

**Published:** 2024-11-02

**Authors:** Yan Yan, Jingjing Lei, Yuqing Huang

**Affiliations:** 1 Key Laboratory of Environment Change and Resources Use in Beibu Gulf, Nanning Normal University, Ministry of Education, Nanning 530001, China; yanyan@nnnu.edu.cn; 2 Guangxi Key Laboratory of Earth Surface Process and Intelligent Simulation, Nanning 530001, China; 3 School of Geographic Sciences and Planning, Nanning Normal University, Nanning 530001, China; leijingjing@email.nnnu.edu.cn

**Keywords:** biomass, UAV LiDAR, regression model, partial least squares regression, *Eucalyptus*

## Abstract

*Eucalyptus* is a widely planted species in plantation forests because of its outstanding characteristics, such as fast growth rate and high adaptability. Accurate and rapid prediction of *Eucalyptus* biomass is important for plantation forest management and the prediction of carbon stock in terrestrial ecosystems. In this study, the performance of predictive biomass regression equations and machine learning algorithms, including multivariate linear stepwise regression (MLSR), support vector machine regression (SVR), and k-nearest neighbor (KNN) for constructing a predictive forest AGB model was analyzed and compared at individual tree and stand scales based on forest parameters extracted by Unmanned Aerial Vehicle–Light Detection and Ranging (UAV LiDAR) and variables screened by variable projection importance analysis to select the best prediction method. The results of the study concluded that the prediction model accuracy of the natural transformed regression equations (R^2^ = 0.873, RMSE = 0.312 t/ha, RRMSE = 0.0091) outperformed that of the machine learning algorithms at the individual tree scale. Among the machine learning models, the SVR prediction model accuracy was the best (R^2^ = 0.868, RMSE = 7.932 t/ha, RRMSE = 0.231). In this study, UAV-LiDAR-based data had great potential in predicting the AGB of *Eucalyptus* trees, and the tree height parameter had the strongest correlation with AGB. In summary, the combination of UAV LiDAR data and machine learning algorithms to construct a predictive forest AGB model has high accuracy and provides a solution for carbon stock assessment and forest ecosystem assessment.

## 1. Introduction

As one of the most extensively cultivated species in the world, *Eucalyptus* spp. plantations have been planted in more than 100 countries because of their fast-growing rate and wide adaptability [1,2,3]. Over the past few years, China has become a major area of *Eucalyptus* plantation due to massive afforestation and reforestation projects [4]. *Eucalyptus* spp. plantations are highly valued as they not only provide industrial timber and pulpwood services but also promote ecosystem services and mitigate climate change by storing carbon [5,6,7]. The correct determination of carbon stock or biomass within a forest stand is essential to forest plantation management operations, such as timber harvest, replanting, and forest inventory [8,9,10]. Carbon stock is mostly commonly referred to as forest biomass. The above-ground biomass (AGB) is mainly the most visible and important carbon pool of the terrestrial ecosystem [11].

The harvest method is the most original method, as all the trees of the forest stand were cut down and weighed after all the components were oven-dried [12,13]. Although this method is considered the most accurate and robust, it is destructive and constrained to a small area due to the time-consuming and labor-intensive field measurements [14]. Therefore, the development of efficient methods for estimating AGB is promoted. At present, the allometric equations are commonly used to predict the AGB [10,15], which were developed using tree dimensions, such as diameter at breast height and tree height. This method is generally suitable for homogeneous forests or plantations with similar stand ages.

The most widely used remote sensing techniques could extend the local measurements based on the aforementioned two methods to large scales. One is the passive remote sensing; the other is the active remote sensing [16]. Various passive sensors, such as Landsat, MODIS, SPOT, QuickBird, and others, operating within the visible to infrared spectrum, have been employed for biomass estimation due to their ability to offer substantial information about the forest canopy layer [11,17,18,19]. However, optical remote sensing is constrained due to its limited capability for penetration [20]. Comparatively, active remote sensing, including synthetic aperture radar (SAR) [21,22] and the Light Detection and Ranging (LiDAR) system [23,24], could penetrate the forest canopy and reach the ground surface. The SAR sensors use microwave backscatter to measure vegetation structure regardless of weather conditions. Nevertheless, the application of both optical and SAR sensors is constrained by the saturation phenomenon, especially in areas with dense vegetation [25,26], whereas saturation of signals is not an issue with LiDAR data. As LiDAR can acquire a three-dimensional profile of the vegetation vertical structure [27], which is strongly related to forest biomass, it has been a promising technology for AGB estimation. Wang et al. mapped the aboveground biomass of mangroves using UAV LiDAR technology, and the results showed that it has great potential [28]. Yue et al. combined UAV LiDAR and mathematical statistical regression models to measure the aboveground biomass of crops. The results showed that VGC-AGB based on human–machine LiDAR data had better results [29]. Zolkos et al. compared the accuracy of biomass estimation using different remote sensing methods (including airborne, satellite, optical, radar, and LiDAR) in more than 70 articles and concluded that LiDAR has higher accuracy than other sensors. The mean multiples R^2^ of aircraft discrete return LiDAR and full return LiDAR (R^2^ = 0.76 and R^2^ = 0.80) are significantly higher than radar and passive optical (R^2^ = 0.50 and R^2^ = 0.59) [30]. According to some comparative studies [31,32,33], LiDAR provided a more accurate estimate of AGB than optical satellite sensors or SAR sensors.

In recent years, unmanned aerial vehicle (UAV) remote sensing platforms have improved the application of LiDAR data for estimating AGB, as UAV-based imaging can generate excellent temporal and spatial resolution data at a lower cost compared to conventional aircrafts [34,35,36]. A variety of methods have been explored by previous studies to establish the correlations between LiDAR parameters and AGB. Generally, there are two kinds of methods, i.e., empirical regression models (e.g., multiple linear regression) and non-parametric (e.g., machine learning) approaches [37,38]. The regression model has been a popular method to estimate vegetation biomass, as it identifies simplified and distinct mathematical relationships between LiDAR metrics and AGB. However, it is restricted by the statistical hypotheses that fail to account for heteroscedasticity, nonlinearity, and multicollinearity problems [39]. Machine learning algorithms like support vector machine (SVM) [18], random forest (RF) [40], and k-nearest neighbors (KNN) [41] have proven to be exceptional techniques for enhancing the estimation accuracy of AGB by leveraging computers’ capabilities in data mining to identify intricate associations between LiDAR-derived metrics and AGB. Therefore, the selection of appropriate metrics as input variables is critical for machine learning algorithms.

Individual tree parameters (including tree height, crown width, DBH, density, number category, etc.) used to estimate AGB can be directly extracted from individual tree data obtained by LiDAR. Stand parameters (canopy density, cover, leaf area index, etc.) are obtained from point cloud structure features, among which height variables and height percentiles are obtained by the cloud quantile method [42]. Many characteristic variables can be extracted from LiDAR data, which shows that it is necessary to solve the multicollinearity problem between variables and screen out the significantly influential problems. Partial least squares regression is an effective method for solving multivariate problems of predicting variables [43]. Its basic principle is to find the optimal linear relationship between the independent variable and the dependent variable so as to obtain the principal component data of the characteristic variable and the correlation coefficient between the measured AGB and the principal component and then use the variable importance in projection (VIP) [44] formula to calculate the importance of the characteristic variable. Variables with significant influence are screened out by importance threshold, and those greater than the set threshold are considered significant variables. The selection of suitable variables from LiDAR data and the accuracies of estimation models for specific studies are varied with forest types [45,46,47]. As a result, the optimal algorithm for estimating forest AGB has yet to be determined [48,49], and it is crucial to make full use of abundant data to accurately and efficiently estimate AGB over a large area with high precision.

For the purpose of improving the management and productivity of the *Eucalyptus* plantation, the goals of this study are (1) to obtain individual tree-level parameters after segmentation to estimate the AGB based on a multivariate linear regression model; (2) to figure out the most significant variables by partial least square regression and establish three machine learning models for AGB estimation; and (3) to compare the performance of the AGB estimation models and screen the optimal models for *Eucalyptus* plantation by leave-one-out cross-validation. The AGB in the study area was mapped and predicted using the best-performing model.

## 2. Material and Methods

### 2.1. Study Area

**Figure 1:**
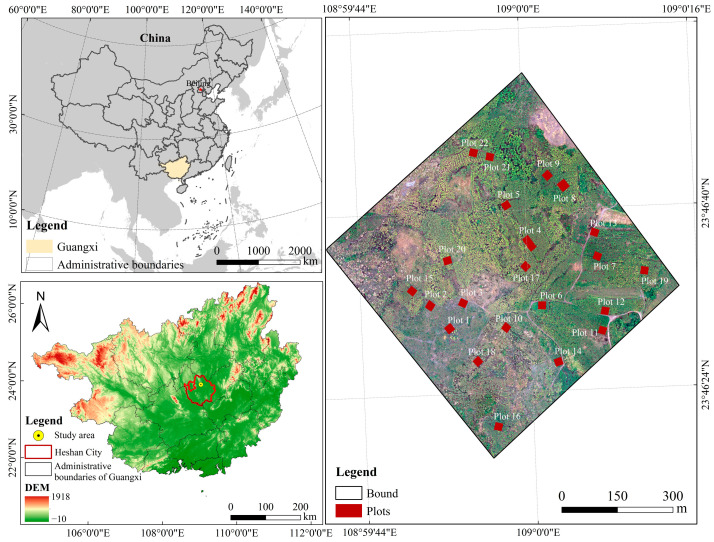
Location of the study area.

The study area (108°59′59″ E, 23°46′36″ N) is situated in the western region of Laibin City within the Guangxi Zhuang Autonomous Region (Figure 1), with a land area of roughly 0.49985 km^2^. The study area is mainly covered by plantation forestry, with *Eucalyptus* spp. being the dominant species, presented a homogeneous forest structure. The region exhibits a subtropical monsoon climate, marked by ample sunshine and copious rainfall. Annual precipitation levels range from 1344 mm to 1460 mm, with the majority occurring during the summer season. The average annual temperature stands at 20.7 °C, with July being the hottest month with a mean temperature of 28.6 °C, and January being the coldest month with a mean temperature of 10.9 °C. In terms of geomorphology, the study area is characterized by karst landforms and has a relatively flat slope with an altitude range of 208 m to 301 m.

The UAV used in this study was a customized product produced by Beijing Digital Green Earth Technology company Limited by Share Ltd. (Beijing, China), such that there was no specific model. The LiDAR sensor was LiDA1350, which could cover a wide range of 6–8 km with a point cloud density of more than 40 points/m^2^. The specific parameters of the UAV LiDAR data include flight altitude of 60 m, flight speed of 3.2 m/s, flight direction of 45 degrees north by east, heading overlap of 80%, and lateral overlap of 40%.

Firstly, the individual tree segmentation was performed for plots 1–10, and LiDAR individual tree parameters were extracted to prepare for the subsequent estimation of AGB. Then, all plots (22 plots) were used as data for the AGB prediction models. Twenty-two square (20 m × 20 m) sample plots were randomly established and discreetly distributed in the study area, except for plot 4 (20 m × 40 m). As *Eucalyptus* is a short-rotation timber forest with a 5-year rotation, the selection also covered different stand ages of the study area. We only measured the trees with diameters at breast height (DBH) greater than 2.5 cm in the sample plots. DBH was calculated by measuring the circumference of the trunk with a tape measure at a height of 1.3 m above the ground. The structural parameters, including height and crown size, were also measured and recorded (Table 1). Each tree’s height was measured twice with an SRC-1/30 height finder, and the average was recorded. The crown size was calculated using the projection method after measuring the length of the canopy projection to the ground with a measuring tape. The individual tree position was also measured by a real-time kinematic global positioning system. These data were used to evaluate the accuracy of the tree positions detected by the different tree segmentation methods examined in this study.

### 2.2. Lidar Data Collection and Pre-Processing

A combination of Lidar data and high spatial resolution orthophoto images was collected in October 2019 during the same period that field measurements were implemented. The UVA vehicle performed the flight 60 m above the ground at a speed of 3.2 m/s with a northeast 45° flight direction. A discrete return laser pulse is used in this system, and the first and last returns per pulse are recorded. The average point density is appr. 300 points/m^2^, and the data were deposited using the CGCS2000 coordinate system as LAS format.

LiDAR360 software 7.0 (Green Valley International, Ltd., Merced, CA, USA) was exploited for data preprocessing, which included the following: (1) Removing noise from raw data through the distance thresholding method; (2) Separating ground points and non-ground points from the LiDAR point cloud data using the progressive morphological filtering algorithm [50]. Determining the grid size for segmenting the point cloud based on the discrete point cloud and applying the morphological approach to identify the lowest point of grid as the ground seed point was the fundamental notion behind the progressive morphological filtering algorithm. In combination with the actual conditions of study area (the height of bush was roughly 1.2 m), an irregular triangulated network was constructed from the seed point and encrypted at 1.4 m intervals until all ground points and non-ground points could be identified; (3) Creating a digital terrain model (DTM). The ground points were interpolated utilizing the inverse distance weighted [51] interpolation method based on ArcMap (V 10.8) software.

In order to obtain the normalized point cloud data for further tree segmentation, the ground elevation was subtracted from the original LiDAR elevation using the DTM obtained. The canopy height model (CHM) is generated by rasterizing the normalized point cloud data. The median filtering method was used to optimize the CHM, which preserved the canopy information more completely and retained the absolute elevation information of the trees in the normalized point cloud.

### 2.3. Individual Tree Segmentation

**Figure 2:**
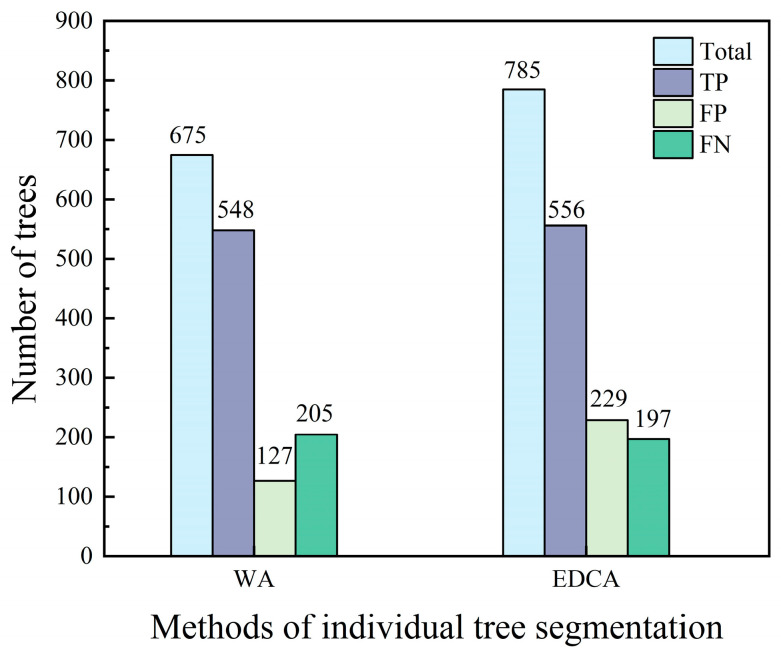
Methods of individual tree segmentation; “Total” denotes total number of individual tree segmentation; “TP” denotes number of true positives; “FP” denotes number of false positives; and “FN” denotes number of false negatives.

Two algorithms were employed to segment the individual tree, including watershed algorithm (WA) [52] and Euclidean distance clustering algorithm (EDCA) [53]. The former is established based on the CHM, while the latter algorithm is based on the normalized point cloud data. The performance of the two segmentation methods was evaluated and compared (Figure 2). The result indicated that while the number of true positives for both approaches is comparable, segmentation accuracy of EDCA was superior to WA due to a significantly higher number of false positives. As a result, the watershed algorithm was employed in the study to segment individual trees.

### 2.4. Characteristic Variables and Importance Analysis

#### 2.4.1. Individual Tree Parameters

Tree height and DBH are the most important parameters for AGB estimation. Although the DBH information is not possible to generate from LiDAR data directly, it is closely related to tree height and crown size. As a result, three parameters were selected to establish the prediction model of AGB. The three parameters were arithmetic mean tree height (AvgHA), weighted average height of crown (LorCHA), and average crown width of trees (CE).

(1) $$\mathrm{AvgHA}=\frac{\sum_{i=1}^{N}{\mathrm{HA}}_{i}}{N}$$

where AvgHA is the arithmetic mean tree height; HA_i_ is the individual tree height; and N is the total number of trees of the stand.

(2) $$\mathrm{LorCHA}=\frac{\sum_{i=1}^{N}{\mathrm{HA}}_{i}\times {\mathrm{CA}}_{i}}{\sum_{i=1}^{N}{\mathrm{CA}}_{i}}$$

(3) $$\mathrm{CA}=\frac{\pi \times {\mathrm{CE}}^{2}}{4}$$

where LorCHA is the weighted average height of crown; CA is the canopy area of individual trees; and CE is the average crown width of trees.

#### 2.4.2. Stand Parameters

The point cloud quantile method was used to extract 35 stand parameters, including tree height variables, tree height percentiles, leaf area index, and other parameters (Table 2). The average values of the stand parameters inside each grid, which were created by dividing the research area into 10 m × 10 m grids, were considered as independent variables to build the AGB prediction models. Then, within the 10 m × 10 m grid, the AGB estimate models were developed by machine learning methods and regression analysis. Additionally, a leave-one-out cross-validation (LOOCV) method was applied to evaluate the prediction models’ performance.

#### 2.4.3. Variable Importance Analysis

The point cloud data contains rich AGB-related feature parameters. According to the crown characteristics and point cloud structure characteristics [42,45,55], 35 potential variables were extracted to construct the AGB prediction models. In this study, the number of features is greater than the samples, and there may be a noticeable correlation between the characteristic parameters. Inputting all features will easily lead to oversaturation and data redundancy and will also affect the prediction accuracy of the model. Therefore, it is necessary to determine the importance of characteristic variables to improve the interpretability of the model.

Partial least squares regression (PLSR) is a statistical method that was developed by combining the advantages of multiple linear regression analysis, canonical correlation analysis, principal component analysis, and other techniques. It is a better method for choosing model parameters and discriminating. Variable importance in projection (VIP) is a measure of the importance of a single independent variable X in a given model in explaining the dependent variable Y. It is the process of variable screening on the principle of the idea of PLSR. In this study, VIP = 0.8, proposed by Wold [56], was used as the critical value for determining significant and non-significant variables. If the importance of the independent variable to the dependent variable is greater, the VIP value will be greater. Otherwise, if the VIP value is less than the critical value, the interpretation of the independent variable to the dependent variable is less.

The importance of the extracted LiDAR-derived variables was analyzed by using this method, from which the derived parameters with greater influence on AGB were screened out. The calculation is as follows:

(4) $$\mathrm{VIP}=\sqrt{\frac{m}{\sum_{i=1}^{n}r^{2}\left(y\right.,\left.a_{i}\right)}\sum_{i=1}^{n}r^{2}\left(y\right.,\left.a_{i}\right)w_{\mathrm{ij}}^{2}}$$

where m is number of LiDAR-derived variables; n is the number of samples; a_i_ is the principle component extracted from LiDAR-derived variables; r (y, a_i_) is the correlation coefficient between the measured biomass of the sample plot and the principal component, which indicates the explanatory ability of the principal component to the biomass of the sample plot y; and w_ij_ is the weight of the variables on the principal component.

### 2.5. Prediction Models of AGB

#### 2.5.1. Linear Regression Model

Linear regression (LR) refers to the linear relationship between a single independent variable or multiple independent variables and dependent variables, and multiple independent variables and multiple dependent variables. It has been widely used because of its simple structure, strong interpretability, and ease of implementation. In this study, linear regression between a dependent variable, y, and multiple independent variables was established as follows [57]:

(5) $$y=\beta_{0}+\beta_{1}x_{1}+\beta_{2}x_{2}+\cdots +\beta_{k}x_{k}+\varepsilon$$

where y indicates measured AGB (t/ha); x_1_, x_2_, …, x_k_ denote the predictor variables, i.e., LiDAR-derived parameters; β_0_ is a constant term; β_1_, β_2_, …, β_k_ denotes the coefficient of the regression equation; and ε is the random error.

#### 2.5.2. Support Vector Regression Model

Support vector regression (SVR) is an extension and application of support vector machines for solving regression problems. Compared with general linear regression, SVR is able to solve the regression problem of high-dimensional features. Its basic principle is to regard all sample data as a class and map the sample points into a high-dimensional space, and then minimize the sum of the errors of the distances of all sample points from the hyperplane by seeking an optimal hyperplane. The equations are as follows [58]:

(6) $$\left|y-f\left(x\right)\right|=\left\{\begin{array}{c} 0\\ \left|y-f\left(x\right)\right|-\varepsilon \end{array}\right.\begin{array}{c} \left|y-f\left(x\right)\right|\leq \varepsilon \\ \left|y-f\left(x\right)\right|>\varepsilon \end{array}$$

where y indicates measured AGB (t/ha); f(x) denotes the prediction model; and ε denotes the tolerance deviation, also known as the error term.

There are several common kernel functions of SVR model (Table 3), and the choice of different kernel functions in the implementation of SVR has an important impact on the regression model. To construct the optimal support vector machine model for predicting AGB, a selection of kernel functions was made, including linear kernel functions, polynomial kernel functions, Gaussian kernel functions, and Sigmoid kernel functions. Furthermore, the loss function (EPSILON_SVR) was defaulted to 0.1 for four models. Except for the linear kernel function, the remaining kernel function has a gamma coefficient of 0.1, a polynomial kernel function of order 2, and a logistic vector scale of 0.01.

The biomass prediction models were constructed based on the featured variables extracted from the UVA-LiDAR data and the measured biomass of the sample plots. The regression models constructed with different kernel functions were obtained, and then the best kernel function was selected based on the model evaluation indexes.

#### 2.5.3. K Nearest Neighbors Model

KNN is a simple and efficient multivariate nonparametric statistical method that intuitively approximates the correlation between independent variables and successive outcomes by averaging observations in the same neighborhood. The principle of KNN in the estimation of forest AGB is as follows: in the sample feature space, the k closest samples to the sample to be estimated are found by calculating the distance metric, and then the forest AGB of the k samples is calculated by using the distance-weighted average method, which in turn yields the forest AGB of the sample to be estimated. The calculation expression is as follows [41]:

(7) $$\;A_{p}=\sum_{i=1}^{k}W_{p,\mathrm{pi}}\times A_{\mathrm{pi}}$$

where A_i_ indicates the forest AGB of the sample plot to be measured. A_pi_ denotes the forest AGB of a known sample plot p_i_; and W_p,pi_ is the weight, and its calculation expression is shown as Equation (8):

(8) $$\;W_{p,\mathrm{pi}}=\frac{1/D_{p,\mathrm{pi}}^{2}}{\sum_{i=1}^{k}1/D_{p,\mathrm{pi}}^{2}}$$

where D_p,pi_ is the distance between the variables characterizing the sample plot, the most commonly used distance measure is the Euclidean distance. When the data are dense or continuous, using the Euclidean distance metric works best. Therefore, we use the Euclidean distance to calculate D_p,pi_ and its calculation expression is shown as Equation (9):

(9) $$\;D_{\mathrm{pi},p}=\sum_{i=1}^{n}{\left(x_{\mathrm{pi}}-x_{p}\right)}^{2}$$

where x_pi_ is the characteristic variable of the image element where the sample plot is located; and x_p_ is the feature variable of the image element to be estimated.

### 2.6. Assessment Model Accuracy

#### Leave-One-Out Cross-Validation

The leave-one-out cross-validation (LOOCV) method was chosen to demonstrate the model’s performance due to the restricted number of plots in this study (22 plots). This verification method is a special case of k-fold cross-validation, which treats k as n-fold cross-validation when the number of samples is n. In this study, each plot was considered as a training subset, and one of them was selected as a test set, which ultimately examined the degree of fitting between the test set and the corresponding training set.

The remaining training sets were repeated from the above steps, and the mean of the 22 fitting degrees were used as the fitting of the model, and then the performance of the model was evaluated. This method is suitable for small data sets with low deviation, and effectively avoids over-fitting or under-fitting. It is a reliable method to evaluate the performance of the model. The indicators for evaluating the performance of the model include determination coefficient ($R^{2}$), root mean square error (RMSE), and relatively root mean square error (RRMSE). The larger $R^{2}$ and the smaller RMSE indicates the higher prediction accuracy of the model. These indicators are calculated by the following formulas [57]:

(10) $$\;\;R^{2}=1-{\sum_{i=1}^{n}\left(Y_{i}\right.-\left.y_{i}\right)}^{2}/{\sum_{i=1}^{n}\left(Y_{i}-\overset{-}{Y}\right)}^{2}$$

(11) RMSE=∑i=1nYi−yi2n

(12) $$\;RRMSE=RMSE/\bar{Y}$$

where n denotes the number of samples; y_i_ denotes the measured biomass value of the plot for its sample, Y_i_ denotes the predicted value of the model for the i plot; and $\overset{-}{Y}$ denotes the mean measured biomass value of the plot.

## 3. Results

### 3.1. AGB Estimation Based on Individual Tree Parameter

**Figure 3:**
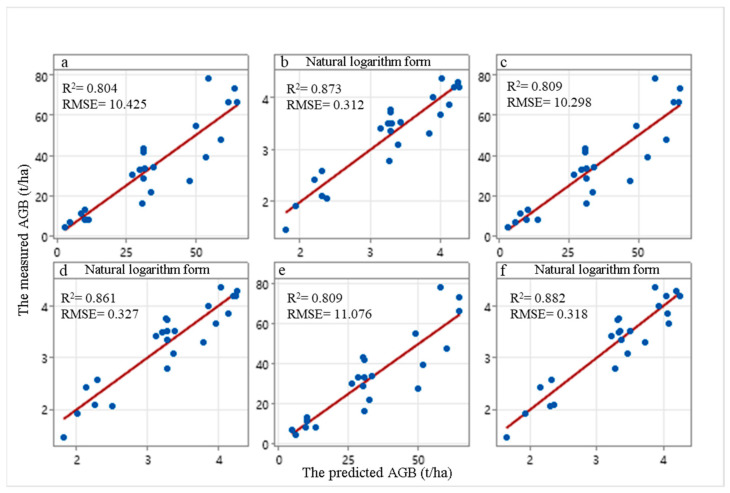
The inversion of forest AGB by LiDAR individual tree parameters; (**a**,**b**) denote the fitting results of single variable AvgHA and measured forest AGB; (**c**,**d**) denote the fitting results of single variable LorCHA and measured forest AGB; (**e**,**f**) denote the fitting results of the two variables (AvgHA and CE) and measured forest AGB.

The linear regression prediction model was established by R 4.2.2 software between the predicted values of individual tree parameters (including estimated arithmetic mean height (AvgHA), crown-weighted mean tree height (LorCHA), and estimated mean crown extent (CE)) and measured forest AGB for total plots, and the model accuracy was tested using leave-one-out cross-validation. The predicted results of forest AGB based on LiDAR individual tree parameters are shown in Figure 3 when the significant level of *p* < 0.05. (b), (d) and (f) denote the AGB fitting of the natural logarithmic model, while (a), (c), and (e) indicate the results using the unnatural logarithmic model. According to the principle that the minimum RMSE of the LOOCV is the optimum [59], the natural logarithmic model has a better fitting. Nevertheless, the results of both models show that AGB was overestimated or underestimated. When AGB was greater than 50 t/ha, the deviation between the biomass points was overestimated or underestimated, and the fitting line was obvious (Figure 3a,c,e). In contrast, the points in the figure are mainly distributed at higher or lower AGB, although there are also deviations, but these are not obvious (Figure 3b,d,f). Figure 3e,f represent the results of the bivariate (AvgHA and CE) fits, but the difference between the bivariate and univariate fitting effects is not obvious.

Linear regression equations were established by LR for the predicted AGB and LiDAR individual tree parameters (Table 4). The fitting degree of the six models was greater than 0.74, indicating that the results were reliable. According to the principle of minimum RMSE and RRMSE, the fitting results of the natural logarithmic transformation model are superior to the direct fitting results. The RMSE decreased from 10.298~11.076 t/ha to 0.312~0.327 t/ha. The regression model constructed based on the natural log transformation of AvgHA (RMSE = 0.312 t/ha, RRMS = 0.0091) is slightly greater than the regression model of AvgHA and CE (RMSE = 0.318 t/ha, RRMS = 0.0092). It can be observed that the contribution of CE to the explanation of AGB is not significant. Although the addition of CE can improve the correlation of the LR model, it also affects the prediction accuracy. Therefore, this study selected the linear fitting equation of univariate AvgHA after natural logarithm transformation to predict forest AGB.

**Figure 4:**
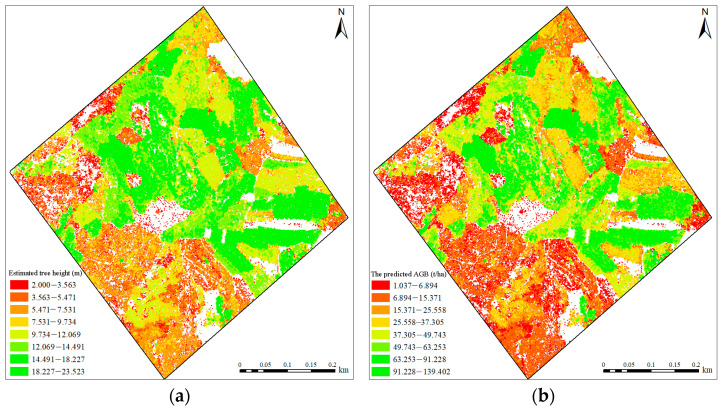
(**a**) The height distribution of individual trees in the study area; and (**b**) the spatial distribution of forest AGB based on individual tree parameters.

The canopy boundary of individual trees segmented by WA was spatially correlated with the LiDAR individual tree segmentation parameters to estimate the tree height of the plots, and the transformed tree height was input into the optimal prediction model to predict the *Eucalyptus* AGB in the study area. Figure 4a,b, respectively, indicate the spatial distribution of the estimated tree height and predicted AGB. It was found that the spatial location of the predicted *Eucalyptus* AGB was related to the predicted tree height. It is noted that the AGB is corresponding to the height of trees. Furthermore, the AGB in high vegetation areas is comparatively large, while the low vegetation area is relatively small, which has a significant spatial correlation. Therefore, tree height is an important individual parameter for predicting forest AGB.

### 3.2. AGB Estimation Based on Stand Parameter

#### 3.2.1. Variable Importance in Projection

**Figure 5:**
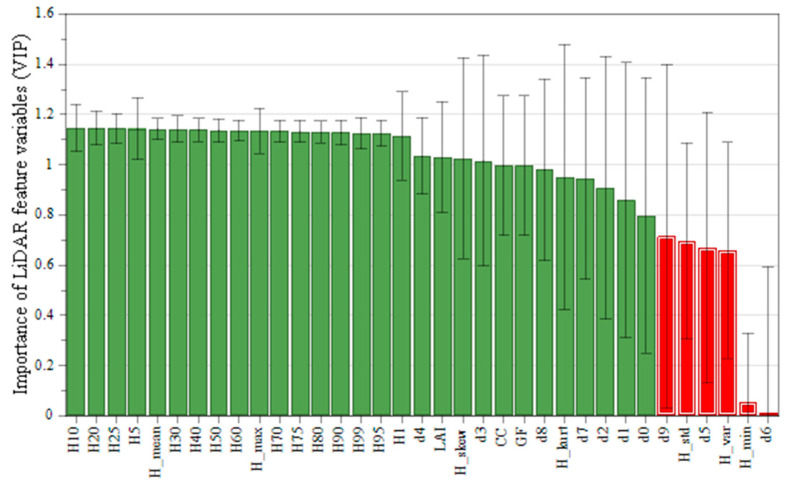
LiDAR features variable importance ranking: green indicates significant characteristic variables, and red indicates insignificant characteristic variables.

Variable importance in projection (VIP) is an effective measure to screen factors or variables, which reflects the relative importance of specific variables in prediction. In this study, SIMCA 17.0 software was used to establish a partial least squares prediction model, and then the optimal feature variable set was selected from the 35 feature variables extracted from the normalized point cloud. The screening threshold is set to 0.8, and the importance is sorted according to the size of the VIP value. Figure 5 shows the screening results, where green indicates significant variables with VIP value greater than or equal to 0.8, while red indicates insignificant variables. When the VIP of the characteristic variable is larger, the correlation between the variable and AGB is stronger. According to Figure 5, 29 variables were selected as important variables and will be used to inverse *Eucalyptus* AGB. Among them, H_10_ showed excellent importance (VIP = 1.15), and the relatively less important was d_0_ (VIP = 0.8). Moreover, the number of height percentiles accounts for 51.7% of the important variables, so the correlation between height percentiles and AGB is the most significant.

#### 3.2.2. Aboveground Biomass Inversion Based on Three Models

(1) Multiple Linear Stepwise Regression Model

In order to improve the prediction result of the models, characteristic variables were further screened using stepwise regression to obtain optimal independent variables after the importance of screening. Based on multiple linear regression, multiple linear stepwise regression (MLSR) introduces the variables into the model one by one. After testing, the meaningless variables are eliminated until the variables in the regression equation no longer change, and the variables are not deleted, thereby obtaining the optimal model with the least variables retained. In this study, SPSS 19.0 software was applied to perform MLSR on the 29 characteristic variables and the measured AGB. Then, LOOCV was utilized to evaluate the performance of the model. The comparison results of model accuracy are shown in Table 5. Specifically, MLSR 1 and 2 are models in which some characteristic variables were removed through MLSR analysis when the impact on the prediction results was acceptable, and the height percentage H10, H10, and LAI were considered predictive variables, respectively.

Table 5 indicates that the H10 and LAI have a higher correlation with the measured AGB than other characteristic variables, and R is greater than 0.8. The goodness of fit of the two optimized models is relatively great, and the R^2^ are higher than 0.75. As the number of variables increases, R, adjusted R^2^, and R^2^ also increase, and RMSE and RRMSE gradually decrease, which demonstrates that the model accuracy of MLSR 2 is superior to that of MLSR 1. Therefore, MLSR 2 was selected to predict the forest AGB, and the equation is as follows:

(13) AGB=−16.324+4.134×H10+27.143×LAI

where AGB is the predicted above ground biomass of forests.

(2) Support Vector Regression Model

**Figure 6:**
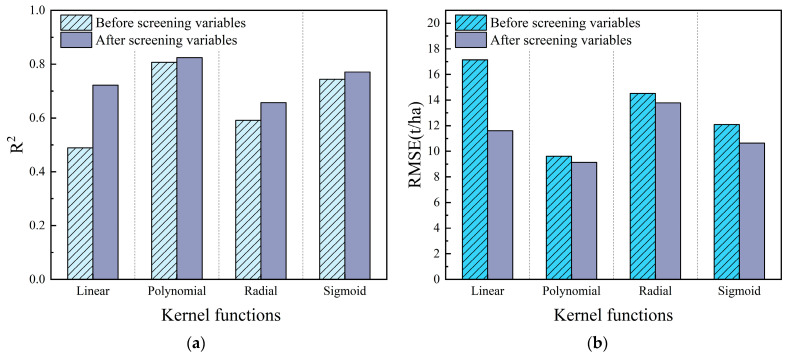
R^2^ and RMSE for different kernel functions in the SVR model: (**a**) is the R^2^ of the four kernel functions before and after variable screening; and (**b**) is the RMSE of the four kernel functions before and after variable screening.

Support vector machine regression (SVR) is a branch of support vector machines that is widely applied in curve regression analysis. We used the e1071 package of R 4.2.2 software to construct the SVR predictive model. Under the four kernel functions, LOOCV is used to evaluate the performance of the model before and after feature variable screening. The SVR forest AGB models constructed by linear kernel function, polynomial kernel function, Gaussian kernel function, and Sigmoid kernel function before and after feature variable screening are displayed in Figure 6.

R^2^ and RMSE were employed as indicators to measure the accuracy of models. As R^2^ is closer to 1 and the RMSE is smaller, it indicates that the regression model performs better. In Figure 6, the fitting and accuracy of the predictive model after variable screening are better than that before variable screening. The maximum difference is 0.168 in R^2^, and the maximum difference is 3.36 t/ha in RMSE, where the polynomial kernel function has the smallest difference in the prediction results before and after the variable screening, while the linear kernel function is the opposite. The results show that the prediction results of the polynomial kernel function regression model are optimal, R^2^ (before: 0.807, after: 0.824) and RMSE (before: 9.614 t/ha, after: 9.139 t/ha) presenting the best results. Consequently, the polynomial kernel function, which screens characteristic variables, was chosen to build the forest AGB prediction model.

(3) KNN Regression Model

**Figure 7:**
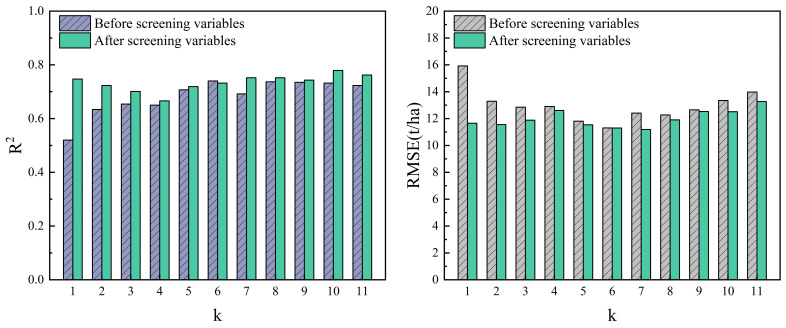
R^2^ and RMSE for different k values in KNN models.

The KNN regression model was built based on the create package of R 4.2.2 software. LOOCV was applied to evaluate the accuracy of the model before and after the variable screening. The k is an important parameter of the KNN model. The performance of the KNN model is different under different k values. Additionally, k is an empirical value and is affected by plot information or forest parameter changes. In previous studies, the range of k is usually set between 1 and 11. Therefore, this study sets k with 1 as the step size and establishes 11 KNN regression models to estimate *Eucalyptus* AGB (Figure 7). The results indicate that the screening variable helps to improve the R^2^ and RMSE of the models. In Figure 7, as k increases, the final (before and after screening variables) trends are similar. When k is between 4 and 10, R^2^ shows an increasing trend, but a downward trend appears after exceeding 10. When k is 4~7, RMSE shows a downward trend and the lowest value. It can be seen that the k should not be too large or too small. According to the principle of minimum RMSE, when k = 7, the error of the k-NN regression model is the smallest (RMSE = 11.191 t/ha), and the prediction result is the best.

#### 3.2.3. Comparison of the Results of Three Models for Inversion of AGB

**Figure 8:**
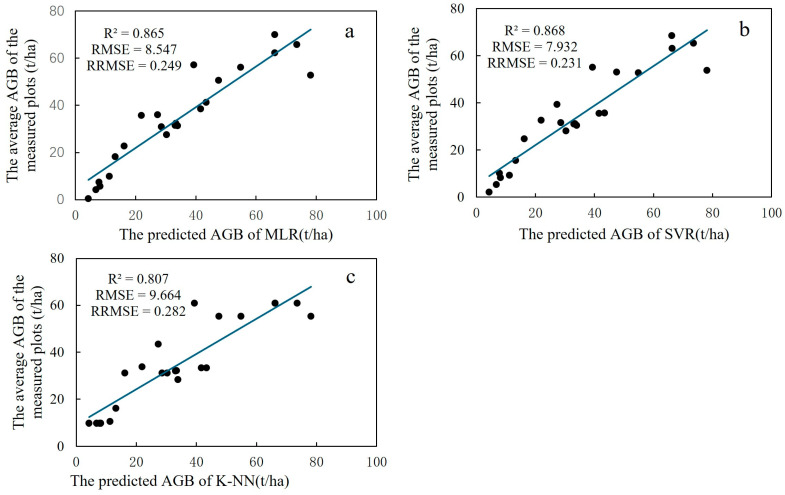
Three models for estimating AGB in forest stands: (**a**) denotes the fitting of the MLR model predicted AGB to the measured AGB; (**b**) denotes the fitting of the SVR model predicted AGB to the measured AGB; and (**c**) denotes the fitting of the KNN model predicted AGB to the measured AGB.

Figure 8 indicates the results of inverting *Eucalyptus* AGB by three optimal models (including MLR, SVR, and KNN), which had higher prediction accuracy but not much difference. In terms of the scatterplot (Figure 8), the three predictive biomass models showed different degrees of overestimation and underestimation. When the *Eucalyptus* AGB is below 50 t/ha, the fitting of the three models is great, and the correlation between the measured value and the estimated value is high. Nevertheless, with the increase in forest AGB, the fitting degree is slightly discrete. By contrast, the prediction accuracy of the SVR model was superior to the other two models, while the SVR model has the best accuracy (R^2^ = 0.868, RMSE = 7.932 t/ha, RRMSE = 0.231), and the inversion results of the KNN model show the most significant overestimation and the worst prediction accuracy (R^2^ = 0.807, RMS = 9.664 t/ha, RRMSE = 0.282).

**Figure 9:**
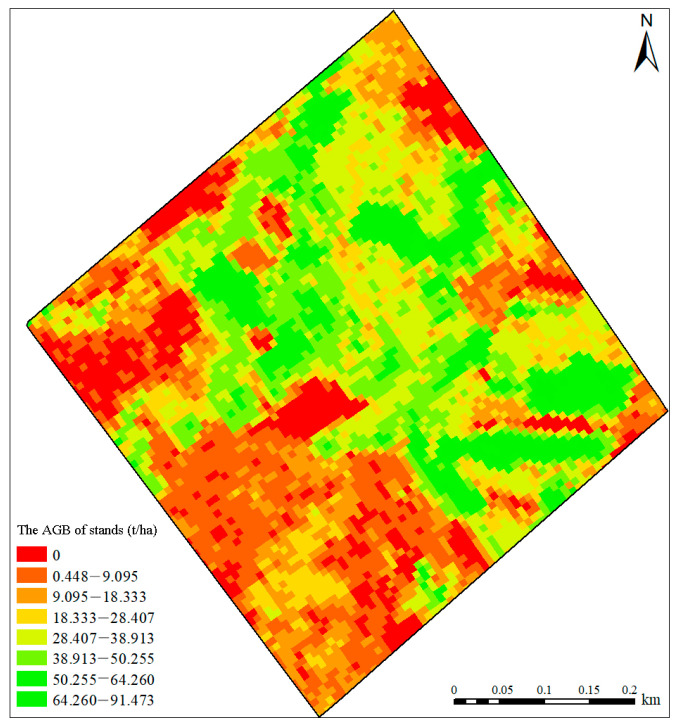
The spatial distribution of the predicted AGB by SVR model.

The spatial distribution of forest AGB in the sample plots was obtained by applying SVR model inversion based on LiDAR feature variables, as shown in Figure 9. The predicted forest AGB densities ranged from 28.407 t/ha to 91.473 t/ha, accounting for about 70% of the study area. The larger AGB were distributed in the eastern and northern parts of the study area, which was consistent with the information from the ground survey. Moreover, the spatial distributions of *Eucalyptus* AGB of different ages were well differentiated. The results interpreted that the SVR model was more applicable in predicting the *Eucalyptus* AGB.

## 4. Discussion

In this study, we screened the optimal characteristic variables of *Eucalyptus* based on UAV LiDAR point cloud data at the individual tree and stand scales, constructed predictive AGB models, and analyzed the prediction accuracy of different models. At the individual tree scale, the naturally transformed AvgHA had the highest accuracy in building the predictive AGB model (R^2^ = 0.851, RMSE = 0.312 t/ha, RRMSE = 0.0091); at the stand scale, the tree height feature parameter was the optimal feature variable, and the SVR predictive model was the most effective (R^2^ = 0.868, RMSE = 7.932 t/ha, RRMSE = 0.231).

### 4.1. Forest AGB Estimation Based on LiDAR Individual Tree Parameters

Inversion of forest biomass by measuring tree height and diameter at breast height (DBH) is a robust method in forest management [60]. However, the ability of UAV LiDAR to acquire tree DBH is restricted [61]. Therefore, tree height and crown spread, which are thoroughly correlated with DBH, were selected for regression fitting with measured AGB in this study. Our results were similar to numerous studies in that the height parameter has a strong correlation with the measured AGB [62,63,64], which is an important indicator for estimating forest aboveground biomass. *Eucalyptus*, the subject of our study, has characteristics of straight trunks and branches concentrated at the top. Therefore, the height variables obtained by applying LiDAR have significant correlations with AGB for this type of forest. On the other hand, the canopy parameters were affected by the accuracy of individual tree segmentation, causing it to be less relevant to the AGB than the height variable. The main reason is the unavoidable phenomenon of over-segmentation or under-segmentation in tree crown identification.

### 4.2. Forest AGB Estimation Based on Stand Parameters

Screening feature variables with significant importance can improve the prediction accuracy of forest AGB and reduce the degree of overfitting, the redundancy of data, and the complexity of the models [65,66,67]. In this study, the partial least squares method was utilized to analyze the variable importance of forest stand parameters (including vertical features of the point cloud, point cloud density features, and canopy features). As in some previous studies, height characteristics are more significantly correlated with the AGB of forests [68,69,70]. The study by Gao et al. [45] supports our view to a certain extent. However, the difference is that this study concluded that point cloud features are also more significantly correlated with the AGB of broadleaf forests. A possible reason is that different tree species have different optimal characterization variables.

The study applied three machine learning methods, MLSR, SVR, and KNN, to predict AGB, respectively. The results concluded that the SVR model had the best estimation, followed by the MLSR model, and the KNN model was the worst. Estimating forest AGB using the SVR method had been a more robust method compared to MLSR [71,72]. Although MLSR eliminates most of the variables with low correlation, which can improve the accuracy to a certain extent, the relationship between the point cloud feature variables and AGB may be nonlinear, which affects the prediction accuracy of the MLSR model. The results of Tang et al. [73] and Fassnacht et al. [26] were basically consistent with our research results. Previous studies have shown that KNN models gave better results than linear regression models based on data flexibility and a data-driven approach [26,74,75]. Unlike previous studies, not all nonparametric algorithms have better prediction accuracy than parametric algorithms [76]. Monne et al. [77] indicated that the multiple regression model established using data optimized by principal component analysis predicts better than the SVR model. In this study, the prediction accuracy of KNN is not as good as MLSR. Because KNN is sensitive to multidimensional data, the dimension of the feature space grows exponentially with the increase in the number of feature variables [31], which can easily lead to misclassification, thus reducing the accuracy of the model.

To summarize, the accuracy of this study in combining UAV LiDAR point cloud data with machine learning algorithms applied to forestry resource surveys is high. However, it is not enough to discuss the adaptability of a single tree species. In addition, this paper does not consider the correlation between terrain feature variables and AGB.

## 5. Conclusions

This study explored the prediction accuracy of different models for *Eucalyptus* AGB based on UAV LiDAR data. In terms of feature variable extraction, the feature variables with the best modeling effect were selected from five types of features: tree height, canopy density, LAI, canopy cover, and gap ratio according to the variable projection importance analysis method. The research results are as follows:

(1) Estimation of forest AGB based on single tree parameters

The three parameters of the LiDAR data (including AvgHA, LorCHA, and CE) were extracted to establish linear regression fitting with the measured AGB. The results indicate that the naturally transformed AvgHA had the best fitting result, R^2^ = 0.851, RMSE = 0.312 t/ha, RRMSE = 0.0091. Since the accuracy of single tree segmentation will affect the accuracy of the crown position, it will affect the fitting results of the crown parameters and the measured AGB. It can be concluded that the AGB of *Eucalyptus* is mainly affected by the tree height parameter.

(2) Estimation of forest AGB based on stand parameters

Thirty-five characteristic variables were extracted based on the cloud characteristics of the sample site. The partial least squares method was used to perform an important projection importance analysis on the characteristic parameters. The results showed that the performance of the fitting model between the selected characteristic variables and the measured AGB was good. The verification determination coefficient (R^2^) and root mean square error (RMSE) of the multivariate linear stepwise regression, support vector machine regression, and k-nearest neighbor model were compared. The results showed that the R^2^ of the three models was greater than 0.8, among which the support vector machine regression model (R^2^ = 0.868, RMSE = 7.932 t/ha, RRMSE = 0.231) had higher accuracy than the other two models. Therefore, it is recommended to use the SVR model to predict the AGB of *Eucalyptus*.

In conclusion, estimating the aboveground biomass of plantations using UAV LiDAR data and machine learning techniques has marvelous applicability and accuracy, making it suitable for use in extensive forest resource assessments. It is worth noting that the study only predicted the AGB of a single tree species in a small area, and the data of single tree segmentation would affect the estimation accuracy of forest biomass. In future studies, the combination of UAV LiDAR and ground-based LiDAR for single tree segmentation will be considered to obtain more accurate point cloud data, and the performance of multiple machine learning regression models in estimating the AGB of different forest types will be discussed.

## Figures and Tables

**Table 1:** Summary of sample plots of the study area.

Variables	Plot 1–10				Plot 11–22			
	Minimum	Maximum	Mean Value	Standard Deviation	Minimum	Maximum	Mean Value	Standard Deviation
DBH (cm)	2.50	15.80	7.76	2.81	3.05	15.9	8.56	3.05
Tree height (m)	3.57	18.00	10.17	3.01	5.05	19.46	11.82	3.48
Crown (m)	1.20	3.80	2.10	0.46	1.08	3.76	2.05	0.44

**Table 2:** LiDAR-derived metrics were considered as candidate variables for AGC estimation.

Metrics	Description
CC (%)	Canopy cover
GF	Gap fraction
LAI (m^2^) [54]	Leaf Area Index
H_kurt	Kurtosis of canopy height
H_max (m)	Maximum height
H_min (m)	Minimum height
H_mean (m)	Mean height
H_skew	Skewness of canopy height
H_std, stddev	Standard deviation
H_var, variance	Variance
H1, H5, H10, H20, H25, H30, H40, H50, H60, H70, H75, H80, H90, H95, H99 (m)	*p*-th percentile of canopy height
d0, d1, d2, d3, d4, d5, d6, d7, d8, d9 (m^2^)	Canopy density variable

**Table 3:** Kernel functions of SVR.

Functions	Expression	Parameters
Linear kernel function	$k\left(x_{i},x_{j}\right)=x_{i}^{T}x_{j}$	$x_{i}^{T}x_{j}$ denotes the inner product of the feature point data
Polynomial kernel function	$k\left(x_{i},x_{j}\right)={x_{i}^{T}x_{j}}^{d}$	d denotes the number of polynomials, d ≥ 0
Gaussian kernel function	$k\left(x_{i},x_{j}\right)=exp\left(-\frac{{\left\|x_{i}-x_{j}\right\|}^{2}}{{2\sigma }^{2}}\right)$	σ denotes the bandwidth of the Gaussian kernel (width), σ > 0
Sigmoid kernel function	$k\left(x_{i},x_{j}\right)=\tan h\left(\beta \right.\left.x_{i}^{T}x_{j}+\theta \right)$	tanh denotes the hyperbolic tangent function, β > 0, θ < 0

**Table 4:** Linear regression equations of measured biomass and LiDAR individual tree segmentation results.

Number	Regression Equations	R	Adjusted R^2^	R^2^	RMSE	RRMSE
1	AGB = 5.201 × AvgHA − 22.213	0.804	0.795	0.77	10.425	0.3043
2	ln (AGB) = 1.99 × ln AvgHA − 1.341	0.873	0.867	0.851	0.312	0.0091
3	AGB = 5.24 × LorCHA − 24.112	0.809	0.80	0.775	10.298	0.3006
4	ln (AGB) = 2.064 × lnLorCHA − 1.581	0.861	0.855	0.837	0.327	0.0095
5	AGB = 4.867 × AvgHA − 6.071*CE − 35.559	0.809	0.80	0.741	11.076	0.3233
6	ln (AGB) = 2.115 × ln AvgHA − 0.8 × ln CE − 0.82	0.882	0.87	0.845	0.318	0.0092

**Table 5:** Summary of multiple linear stepwise regression models.

Model	R	Adjusted R^2^	R^2^	RMSE	RRMSE
MLSR 1	0.816 ^a^	0.807	0.770	9.746	0.284
MLSR 2	0.865 ^b^	0.852	0.822	8.547	0.249

^a^ predictive variable: (constant), H10; ^b^ predictive variables: (constant), H10, LAI.

## Data Availability

The data presented in this study are available on request from the corresponding author.
